# Mud crab susceptibility to disease from white spot syndrome virus is species-dependent

**DOI:** 10.1186/1756-0500-3-315

**Published:** 2010-11-20

**Authors:** Naraporn Somboonna, Seksan Mangkalanan, Attasit Udompetcharaporn, Chartchai Krittanai, Kallaya Sritunyalucksana, TW Flegel

**Affiliations:** 1 Shrimp-Virus Interaction Laboratory, National Center for Genetic Engineering and Biotechnology, National Science and Technology Development Agency, Klong Luang, Pathumthani 12120, Thailand; 2 Center of Excellence for Shrimp Molecular Biology and Biotechnology, Mahidol University, Rama 6 Road, Bangkok 10400, Thailand; 3 Institute of Molecular Biosciences, Mahidol University, Salaya Campus, Nakhonpathom 73170, Thailand; 4 Department of Biotechnology, Faculty of Science, Mahidol University, Rama 6 Road, Bangkok 10400, Thailand; 5 Department of Microbiology, Faculty of Science, Chulalongkorn University, Bangkok 10330, Thailand

## Abstract

**Background:**

Based on a report for one species (*Scylla serrata*), it is widely believed that mud crabs are relatively resistant to disease caused by white spot syndrome virus (WSSV). We tested this hypothesis by determining the degree of susceptibility in two species of mud crabs, *Scylla olivacea *and *Scylla paramamosain*, both of which were identified by mitochondrial 16 S ribosomal gene analysis. We compared single-dose and serial-dose WSSV challenges on *S. olivacea *and *S. paramamosain*.

**Findings:**

In a preliminary test using *S. olivacea *alone, a dose of 1 × 10^6 ^WSSV copies/g gave 100% mortality within 7 days. In a subsequent test, 17 *S. olivacea *and 13 *S. paramamosain *were divided into test and control groups for challenge with WSSV at 5 incremental, biweekly doses starting from 1 × 10^4 ^and ending at 5 × 10^6 ^copies/g. For 11 *S. olivacea *challenged, 3 specimens died at doses between 1 × 10^5 ^and 5 × 10^5 ^copies/g and none died for 2 weeks after the subsequent dose (1 × 10^6 ^copies/g) that was lethal within 7 days in the preliminary test. However, after the final challenge on day 56 (5 × 10^6 ^copies/g), the remaining 7 of 11 *S. olivacea *(63.64%) died within 2 weeks. There was no mortality in the buffer-injected control crabs. For 9 *S. paramamosain *challenged in the same way, 5 (55.56%) died after challenge doses between 1 × 10^4 ^and 5 × 10^5 ^copies/g, and none died for 2 weeks after the challenge dose of 1 × 10^6 ^copies/g. After the final challenge (5 × 10^6 ^copies/g) on day 56, no *S. paramamosain *died during 2 weeks after the challenge, and 2 of 9 WSSV-infected *S. paramamosain *(22.22%) remained alive together with the control crabs until the end of the test on day 106. Viral loads in these survivors were low when compared to those in the moribund crabs.

**Conclusions:**

*S. olivacea *and *S. paramamosain *show wide variation in response to challenge with WSSV. *S. olivacea *and *S. paramamosain *are susceptible to white spot disease, and *S. olivacea *is more susceptible than *S. paramamosain*. Based on our single-challenge and serial challenge results, and on previous published work showing that *S. serrata *is relatively unaffected by WSSV infection, we propose that susceptibility to white spot disease in the genus *Scylla *is species-dependent and may also be dose-history dependent. In practical terms for shrimp farmers, it means that *S. olivacea *and *S. paramamosain *may pose less threat as WSSV carriers than *S. serrata*. For crab farmers, our results suggest that rearing of *S. serrata *would be a better choice than *S. paramamosain *or *S. olivacea *in terms of avoiding losses from seasonal outbreaks of white spot disease.

## Hypothesis

White spot syndrome virus (WSSV) is the world's most serious disease threat to all species of cultivated shrimp, and is also known to infect many other crustacean species that can act as carriers [1,2]. Among these carriers, mud crabs have been considered to be a particularly dangerous threat to shrimp farms because they are generally believed (based on a report for the species *Scylla serrata*) to be highly tolerant to WSSV and remain infected for long periods of time without signs of disease [2-4]. We wished to test this hypothesis by determining the degree of susceptibility and tolerance to WSSV infection in common Thai species of *Scylla *other than *Scylla serrata*. To do so, it was first necessary for us to develop a mitochondrial 16 S rDNA method to distinguish among 4 *Scylla *species found in Thailand, because species determination by morphology alone remains controversial [5-8].

## *Scylla *species identification by analysis of 16 S rDNA sequences

To develop a species identification method based on mitochondrial 16 S rDNA sequences, reference sequences of *S. serrata *(GenBank Accession Number AF109318), *S. paramamosain *(AF109319), *S. olivacea *(AF109321) and *S. tranquebarica *(AF109320) were used to design primers for polymerase chain reaction (PCR) analysis and sequencing. The PCR primer 16sar-L (5' cgcctgtttatcaaaaacat 3') was designed by Imai et al. [9], and primer 16sar-R (5' ggtctgaactcagatcacgt 3') was designed in this study. Each PCR reaction consisted of 10×PCR buffer, 0.75 mM MgCl_2_, 0.1 mM dNTPs, 0.15 μM of each primer, 2.5 U taq DNA polymerase and 100 ng DNA in a total volume of 100 μl reaction. The thermocycling profile was 2 min at 94°C followed by 30 cycles of 94°C for 1 min, 45°C for 30 sec and 72°C 1.5 min. Sequencing was performed by Macrogen Inc., Seoul, Korea. Each sequence was confirmed manually via electropherogram analysis (BioEdit 7.0.0, Carlsbad, CA) [10].

**Figure 1:**
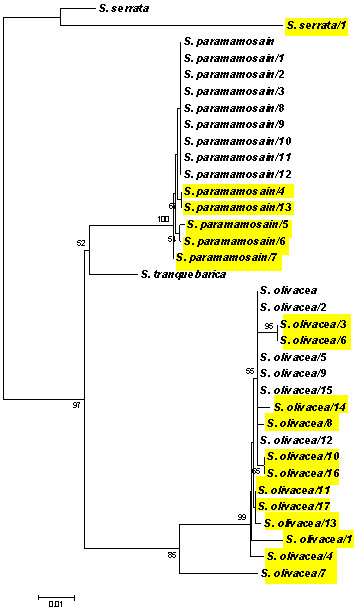
**Phylogenetic relationship of 31 representative *Scylla *specimens from Samutprakarn and Samutsongkram provinces of Thailand**. A neighbor-joining tree was constructed using mitochondrial 16 S rDNA sequences of reference *S. serrata*, *S. paramamosain*, *S. tranquebarica *and *S. olivacea*, and 31 recent *Scylla *species collected from Samutprakarn and Samutsongkram provinces of Thailand. Yellow highlights indicate crabs with at least 1 nt difference compared with those of the respective reference species. The value at each node is the percent bootstrap confidence calculated from 1000 bootstrap resamplings. Bootstrap values of 50% and below have been omitted in the figure. Each branch distance corresponds to sequence divergence.

This method was used with 31 *Scylla *samples arbitrarily collected from 500 g male mud crabs from Samutprakarn and Samutsongkram provinces, Thailand, during 3 different months in 2008. Since the Ethical Principles and Guidelines for the Use of Animals of the National Research Council of Thailand (1999) apply to vertebrates only and there is no official standard for invertebrates, we adapted its principles to crabs. We also followed the guidelines of the Australian, New South Wales state government for the humane harvesting of fish and crustaceans http://www.dpi.nsw.gov.au/agriculture/livestock/animal-welfare/general/fish/shellfish with respect to details regarding the transport of the crabs and their laboratory maintenance. With respect to processing the crabs for histological analysis or for killing at the end of an experiment, the salt water/ice slurry method was used as recommended in the Australian guidelines. The amplicon sizes obtained using our 16 S rDNA PCR method were 562 base pairs (bp) for *S. olivacea*, *S. serrata *and *S. paramamosain *and 563 bp for *S. tranquebarica*. These sequences together with the corresponding regions from GenBank records of *Scylla *species (above), were compared using MEGA 3.1 software [10,11]. Pairwise distances were calculated by the p-distance model using the program default parameters, including different substitution rates for transitions and transversions and a uniform rate of substitution among sites [12]. Pairwise % nucleotide identities and p-distances indicated a close relationship between *S. paramamosain *and *S. tranquebarica *(95.7% nt identity and 0.041 p-distance), whereas *S. olivacea *and *S. serrata *showed a relatively lower percent nt identity and the greatest p-distances (90.8% nt identity and 0.086 p-distance) to the other species. Neighbor-joining trees calculated using the Kimura-2-parameter model and assuming constant nucleotide frequencies and rates of substitution among sites [12,13] revealed 4 clusters that clearly corresponded to the 4 species (Figure 1). Bootstrap replicates (= 1,000) were used to determine the percent confidence for each clustering branch. The topology for any branch with a bootstrap value of 95% or higher is considered true clustering [11]. *S. tranquebarica *was not found in our 31 samples while there were 17 *S. olivacea*, 13 *S. paramamosain *and only 1 *S. serrata*. Since there was only one specimen of *S. serrata*, it was not included in the ongoing tests with WSSV.

## WSSV stock inoculum and virulence confirmation in shrimps

WSSV inoculum was kindly provided by the Shrimp Cultivation Research Center, Charoen Pokphand Group, Thailand. Number of WSSV copies was determined by real-time PCR (qPCR). For quantitation of WSSV stock and WSSV in the samples, DNA (100 ng) were used as the template in a total 20 μl reaction mixture containing 1× QuantiTect SYBR Green PCR Master Mix (Qiagen, the Netherlands) and 300 nM of each primer for qPCR using ABI 7500 SDS machine (Applied Biosystems, Foster City, CA, USA). Primers 229F1 (5' gatggaaacggtaacgaatctgaa 3') and 447R1 (5' cagagcctagtctatcaatcat 3') were designed from the WSSV genome (GenBank Accession No. AF440570) [14]. Each qPCR plate contained standard curve samples, triplicates of each DNA sample, and a no-template control. The thermocycling profile was 95°C for 15 min, followed by 40 cycles of 95°C for 15 s, 55°C for 30 s and 72°C for 45 s [14]. For all experiments, the specificity of the amplified products was verified by analyses of the dissociation curves to verify the melting temperature for each amplicon. The quantity was determined from the standard curve of Ct values and WSSV copy numbers. Results were expressed as mean copy numbers ± standard deviations (SDs) for triplicate samples.

For semi-quantitative estimation of WSSV copy numbers, the IQ2000™WSSV Detection and Prevention System (Farming IntelliGene Technology Corporation, Taipei, Taiwan) was used. Using 100 ng of total DNA as the template, infections could be classified as absent, very light, light, moderate and severe based on 1.5% agarose gel electrophoresis patterns of PCR amplicons, and these were approximately equal to the following respective WSSV copy numbers in the 100 ng template: 0 to <10, 10 to <20, 20 to <200, 200 to <2,000 and 2,000 to <20,000.

To measure WSSV loads in shrimp, hemolymph was withdrawn from the ventral sinus into a syringe containing anticoagulant I (ACI) (0.45 M NaCl, 0.1 M glucose, 30 mM Na-citrate, 26 mM citric acid, 10 mM EDTA, pH 7.0) [15] in a 1:2 volume-to-volume ratio. DNA was extracted following the manufacturer's protocols using a DNeasy Blood & Tissue Kit (Qiagen, Valencia, California, USA). The DNA concentration and quality were measured by spectrophotometry at A_260 _and A_280_, and the amount of WSSV in the samples was determined as described above.

To verify virulence of the WSSV stock, 2 specific pathogen-free (SPF) whiteleg shrimp *P. vannamei *and 6 SPF black tiger shrimp *P. monodon *were injected with 5 × 10^6 ^copies/g tissue at the first abdominal segment. Shrimp mortality for *P. vannamei *and *P. monodon *was 50% and 100%, respectively, within 3-4 days after injection and moribund shrimp gave IQ2000 test results for severe WSSV infection levels. Matching qPCR results ranged from 2 × 10^4 ^- 1.3 × 10^6 ^copies/100 ng DNA (data not shown). This was equivalent to approximately 2 × 10^4 ^- 1.3 × 10^6 ^WSSV copies in 33.33 μl of infected shrimp hemolymph.

These results were similar to those previously published for these and other species of penaeid shrimp that usually show 100% mortality within 5-10 days after injection with similar doses of WSSV [16].

## Preliminary, single-dose challenge with *Scylla olivacea*

Because injection is considered an effective route of WSSV infection in crabs [4], different WSSV copies per gram of crab tissue in a total sterile phosphate buffer saline (PBS) volume of 300 μl was injected into each crab at the coxa of the right swimming leg using a 26G1 syringe (Nipro Corporation Ltd.). A preliminary single-dose challenge test with 34 male *S. olivacea *was carried out to determine appropriate viral challenge doses for crabs. They were divided into three groups. One group (n = 9) was injected with a single dose of 1 × 10^5 ^WSSV copies per g, one group (n = 13) with 1 × 10^6 ^WSSV copies per g and one control group (n = 12) with buffer solution.

At the low injection dose (1×10^5^), 4 of 9 (44%) died within 7 days while 5 of 9 (56%) survived for more than 30 days. However, at a higher dose (1×10^6^), 6 of 13 died on day 3 post injection (46% mortality), 4 more died on day 4 (77% cumulative mortality), 2 more on day 5 (92% cumulative mortality) and 1 on day 7 (100% cumulative mortality in 7 days). None of the 12 buffer-injected crabs died over the 30 day experimental period. These results indicated that a single, high dose challenge with the WSSV inoculum was lethal for *S. olivacea *as it was for shrimp. This contrasted markedly with results from a previous report in which *S. serrata *challenged with WSSV showed severe WSSV lesions by histopathological analysis but no mortality [1,3,17].

## Serial-dose challenge protocol

Male mud crabs (500 g) were collected from Samutprakarn and Samutsongkram provinces, Thailand, and species was determined by 16 S rDNA analysis as described above. Each crab was free of WSSV, yellow head virus (YHV) and infectious hypodermal and hematopoietic necrosis virus (IHHNV) as determined using standard commercial kits for nested PCR and RT-PCR detection (IQ 2000 detection system, Gene Reach, Taiwan). Individual crabs were cultivated in separate plastic containers at room temperature with aeration [18]. Every 24-30 h, crabs were fed with boiled fish at 10% of their body weight, and artificial seawater at 28 parts per thousand (ppt) salinity was replaced to the level where the crabs still had some area to come out of the water [1,18].

For the challenge tests with each species, 18 crabs were acclimatized for 1-2 weeks after which it was expected that 10-12 each would be used for WSSV challenge and 5-6 each for PBS buffer-injected controls. However, since only 30 (not 36) remained in sufficiently good health for testing at the end of acclimatization (17 *S. olivacea *and 13 *S. paramamosain*), they were divided into 20 crabs (11 *S. olivacea *and 9 *S. paramamosain*) for challenge with WSSV, and 10 (6 *S. olivacea *and 4 *S. paramamosain*) as controls. They were numbered sequentially for each species (So 1 to 17 and Sp 1 to 13) but selected arbitrarily for inclusion in challenge or control groups. WSSV challenge began with 1 × 10^4 ^WSSV copies/g at day 0 followed every 14 days by increasing doses of 1 × 10^5^, 5 × 10^5^, 1 × 10^6 ^and ending with 5 × 10^6 ^WSSV copies/g at day 56. The crabs were observed for further 50 days after the final challenge dose (a total of 106 days for the whole challenge experiment).

For these tests, all the buffer-injected control crabs were healthy as defined by no appetite loss, no weight loss, reactive swimming and walking legs, and no mortality throughout the experimental period (106 days) plus the period of 4-10 days during which the crabs were acclimatized, screened for specific pathogens and subjected to PCR and sequencing for species identification. None of the controls for either species gave positive results for WSSV using the IQ2000 test kit, qPCR or histological analysis. For the test crabs given multiple challenges with WSSV, there were generally three types of outcomes as follows: (i) moribund at low dose (< 10^6 ^copies/g), (ii) moribund at high dose (5×10^6 ^copies/g), and (iii) survival at high dose (5×10^6 ^copies/g).

To quantify WSSV in each crab by qPCR or test kit (see above), hemolymph was withdrawn at the coxa of the right swimming leg using a 26G1" syringe (Nipro Corporation Limited, Bangkok, Thailand) containing the same anticoagulant used for shrimp above. DNA extraction was also carried out in the same manner.

## Serial-dose challenge with *Scylla olivacea*

**Figure 2:**
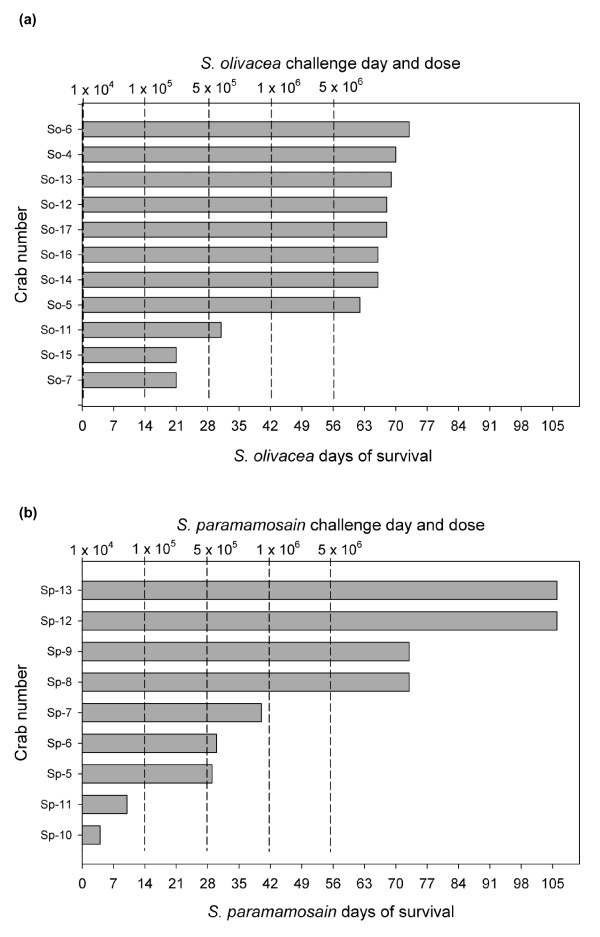
**Survival of *S. olivacea *(a) and *S. paramamosain *(b) after multiple WSSV challenges**.

Of the 11 *S. olivacea *specimens used for the serial-challenges with WSSV (Figure 2a), there was no mortality in 14 days following the initial 1 × 10^4 ^challenge dose, while 2 specimens died 7 days following the 2^nd ^challenge dose (1×10^5 ^copies/g) and 1 died 3 days following the third challenge dose (5×10^5 ^copies/g). Curiously, no mortality occurred during the 2-week period following the dose of 1 × 10^6 ^copies/g, despite the fact that the dose was 100% lethal in the preliminary single challenge test with this species. All the remaining 8 crabs died within 7 to 17 days following the 5^th ^(highest) challenge dose (5×10^6 ^copies/g). The longest survival was for one specimen at 73 days after the initial challenge and the mean time to death was 55.9 ± 20.6 days and the Kaplan-Meier survival probability [19] was 0.63 for 63 days and 0 for 70 days. For this species, the majority of the crabs fell into two rough groups, one that showed mortality after a relatively low challenge dose of WSSV and one that showed mortality only after the highest dose (5×10^6 ^copies/g).

**Figure 3:**
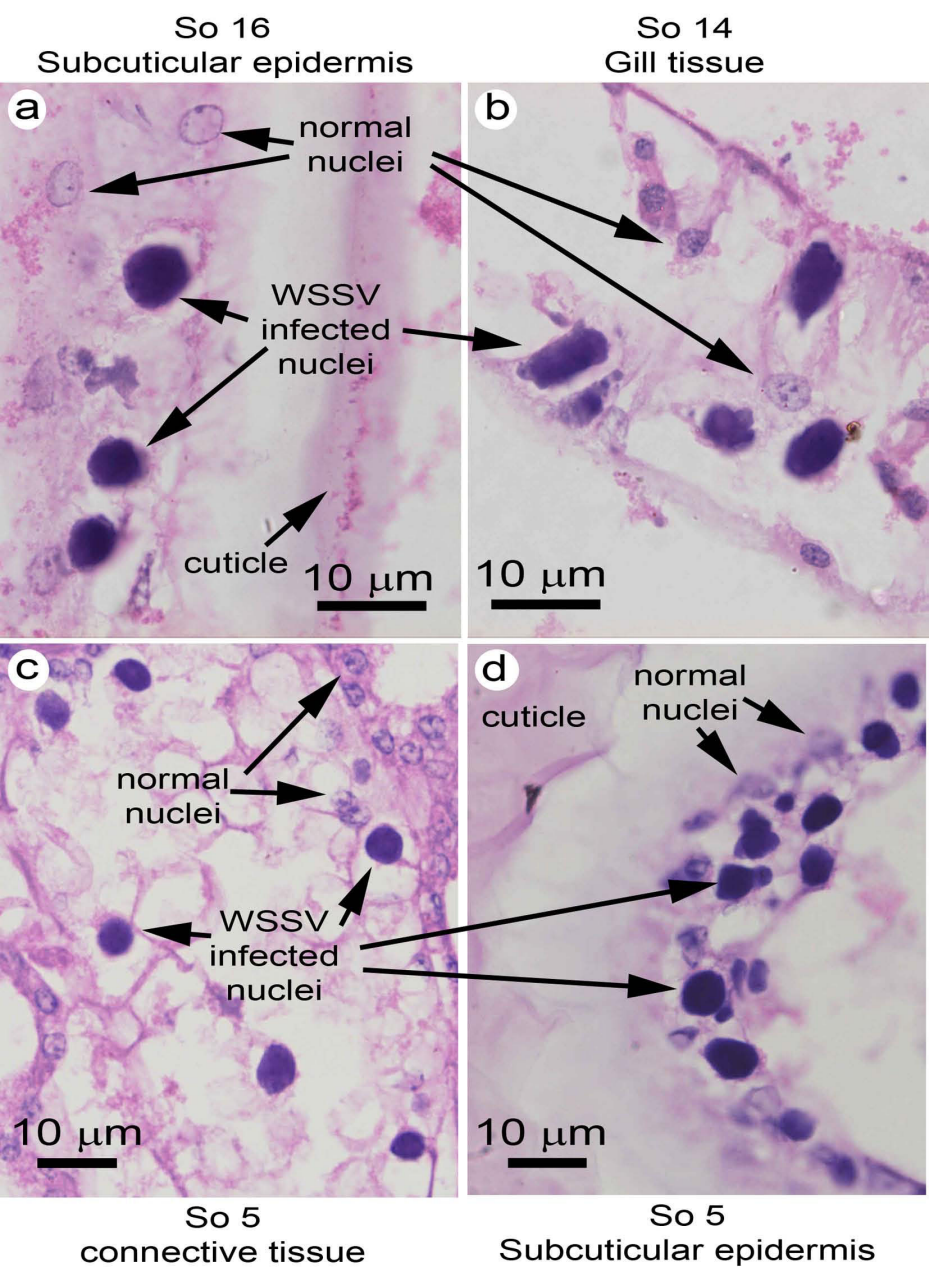
**Histology of *S. olivacea *challenged with WSSV**. Examples of typical WSSV lesions at high magnification in various tissues, indicating heavy infections consistent with PCR test kit and real-time PCR results.

All the challenged *S. olivacea *gave positive reactions for severe WSSV infections using the IQ2000 test kit, except for the 2 crabs that died after the lowest challenge dose of 1 × 10^5 ^copies/g. These gave negative results with the kit. Their WSSV copies were also below the qPCR detection limit. For 8 of the 9 IQ2000 test kit-positive moribund samples, results corresponded to those for qPCR where viral levels ranged from 3.1 × 10^7 ^to 3.4 × 10^9 ^WSSV copies per 100 ng total DNA (mean 6.5 × 10^8 ^copies per 100 ng total DNA). Extracted DNA of 100 ng represented a fresh muscle tissue weight of approximately 0.15 mg, so qPCR results would have to be multiplied by 6.7 × 10^3 ^to obtain viral loads per g fresh crab tissue. Curiously, one sample (So6) gave a reaction for severe infection with the test kit but a qPCR result for only 7 × 10^3 ^WSSV copies per 100 ng total DNA. Of the 9 specimens that gave results for severe reactions with the kit, 7 were examined histologically and all were positive with typical WSSV lesions (Figure 3) except for So6.

**Figure 4:**
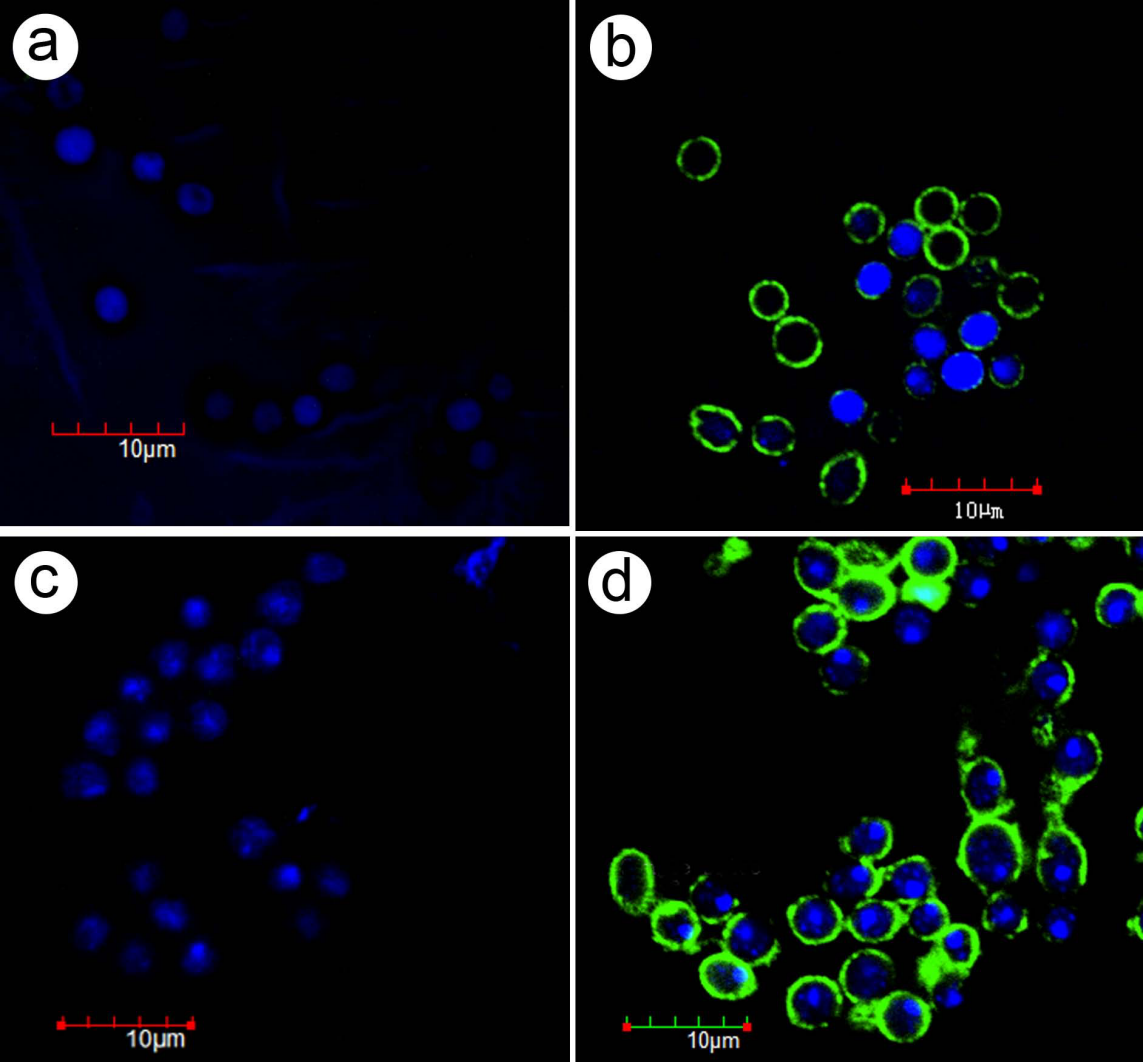
**Immunohistochemical analysis of crab hemocytes**. (**a**) Hemocytes of a normal *S. olivacea *specimen showing large (non-condensed) nuclei. (**b**) Hemocytes of *S. olivacea *(So5 day 59) infected with WSSV showing positive immunofluorescence (green) for WSSV in the cytoplasm. (**c**) Hemocytes of normal *S. paramamosain *specimen showing large (non-condensed) nuclei. (**d**) Hemocytes of *S. paramamosain *(Sp12 day 82) showing positive immunofluorescence for WSSV in the cytoplasm and condensed and fragmenting nuclei.

In addition, hemocytes of 2 samples (So5 and So13) were examined by confocal microscopy for the presence of WSSV by immunohistochemistry. Hemolymph was collected in 1:1 vol./vol. of hemolymph fixative (0.45 M NaCl and 4% formalin in sterile distilled water) and then further fixed with 4% paraformaldehyde in PBS, and permeabilized with 0.1% Triton X-100 (Acros Organics, Morris Plains, NJ, USA) for immunofluorescence analysis using a polyclonal antibody raised against WSSV recombinant VP28 (rVP28) envelope protein. Detection was achieved using a secondary antibody labeled with Alexa Fluor 488 (Molecular Probes, Eugene, OR, USA) as previously described [20]. Negative controls included hemocytes from buffer-injected crabs stained with the anti-WSSV polyclonal antibody, hemocytes from infected crabs with no anti-WSSV polyclonal antibody label, and hemocytes from infected crabs that were stained with a polyclonal antibody raised against monodon baculovirus (MBV), which is not known to infect crabs. TO-PRO-3 (Molecular Probes) was used for nucleic acid counterstaining. Immunofluorescent-labeled cells were analyzed by an FV1000 confocal laser scanning microscope (Olympus, Tokyo, Japan). Results showed immunopositive signals in the cytoplasm for the majority of the hemocytes (Figure 4b).

The multiple challenge group at the highest challenge dose also died, gave test kit results for severe WSSV infections and also showed severe histopathology typical of WSSV. More difficult to explain is the mortality of the 2 *S. olivacea *specimens that died after an injection dose of 1 × 10^5 ^or fewer WSSV copies per g and gave negative results for WSSV by both test-kit assay and qPCR. We assume that they died as a result of some undetermined complication resulting from the WSSV injection, but not because of WSSV alone and not because of the injection process itself, since none of the control crabs died after buffer injections. Similar examples occurred with *S. paramamosain *(below). Most of the *S. olivacea *specimens were not examined histologically because the few examined gave consistent results for severe infections by histopathology and qPCR. We cannot explain the disparity between the test-kit result for severe WSSV infection and the qPCR result for a low WSSV load in specimen So6.

In summary, the overall results suggested that *S. olivace *was generally as susceptible to mortality from WSSV as *P. vannamei *and *P. monodon*. However, the fact that approximately 50% of the *S. olivacea *specimens died within 3 days in the single dose challenge at 1 × 10^6 ^copies of WSSV per g but that 7 of 11 (63.64%) survived for 2 weeks after the same dose in the multiple challenge test indicates that a prior infection with WSSV could aid in the ability of the crabs to better survive a subsequent challenge. This is not surprising in the light of the fact that shrimp survivors from WSSV outbreak ponds have been shown to survive a WSSV challenge dose sufficient to kill uninfected shrimp [21,22], and that prior injection of shrimp with inactivated WSSV can result in improved survival upon subsequent challenge [23]. *S. olivacea *would be a good model for a molecular study on the comparative response of multiple-challenge crabs that survive the WSSV dose (1 × 10^6 ^copies/g) that was sufficient to kill naïve crabs.

## Serial-dose challenge with *Scylla paramamosain*

Of 9 *S. paramamosain *specimens used for serial WSSV challenges (Figure 2b), 2 specimens died within 2 weeks after the 1^st ^challenge dose (1×10^4^). There was no additional mortality in the 2-week interval after the 2^nd ^challenge dose (1×10^5^), but 2 specimens died within 2 days and 1 specimen within 12 days after the 3^rd ^challenge dose (5×10^5^). As with *S. olivacea*, no subsequent mortality occurred within two weeks after challenge with a dose of 1 × 10^6 ^copies/g. Then, 2 specimens died within 17 days after the 5^th ^and highest challenge dose (5×10^6^), while the two remaining crabs were still alive and active at the end of the experiment on day 106. The mean time to death for those that died was 37.0 ± 27.5 days, and this was not significantly different (p = 0.11) from that for *S. olivacea *(55.9 ± 20.6). The Kaplan-Meier survival probability of *S. paramamosain *for 63 days was 0.44 (0.63 for *S. olivacea*) but for 70 to 106 days was 0.22 (0 for *S. olivacea*). Similar to *S. olivacea*, there was one group of crabs that died after low challenge doses and one (22.22%) that died after the highest challenge dose. Unlike *S. olivacea*, there was an additional group (22.22%) that did not die, even at the highest challenge dose. In addition, *S. paramamosain *showed a pattern of proportionally higher mortality at low WSSV doses and proportionally lower mortality at high doses, a pattern that was opposite to that for *S. olivacea*.

**Figure 5:**
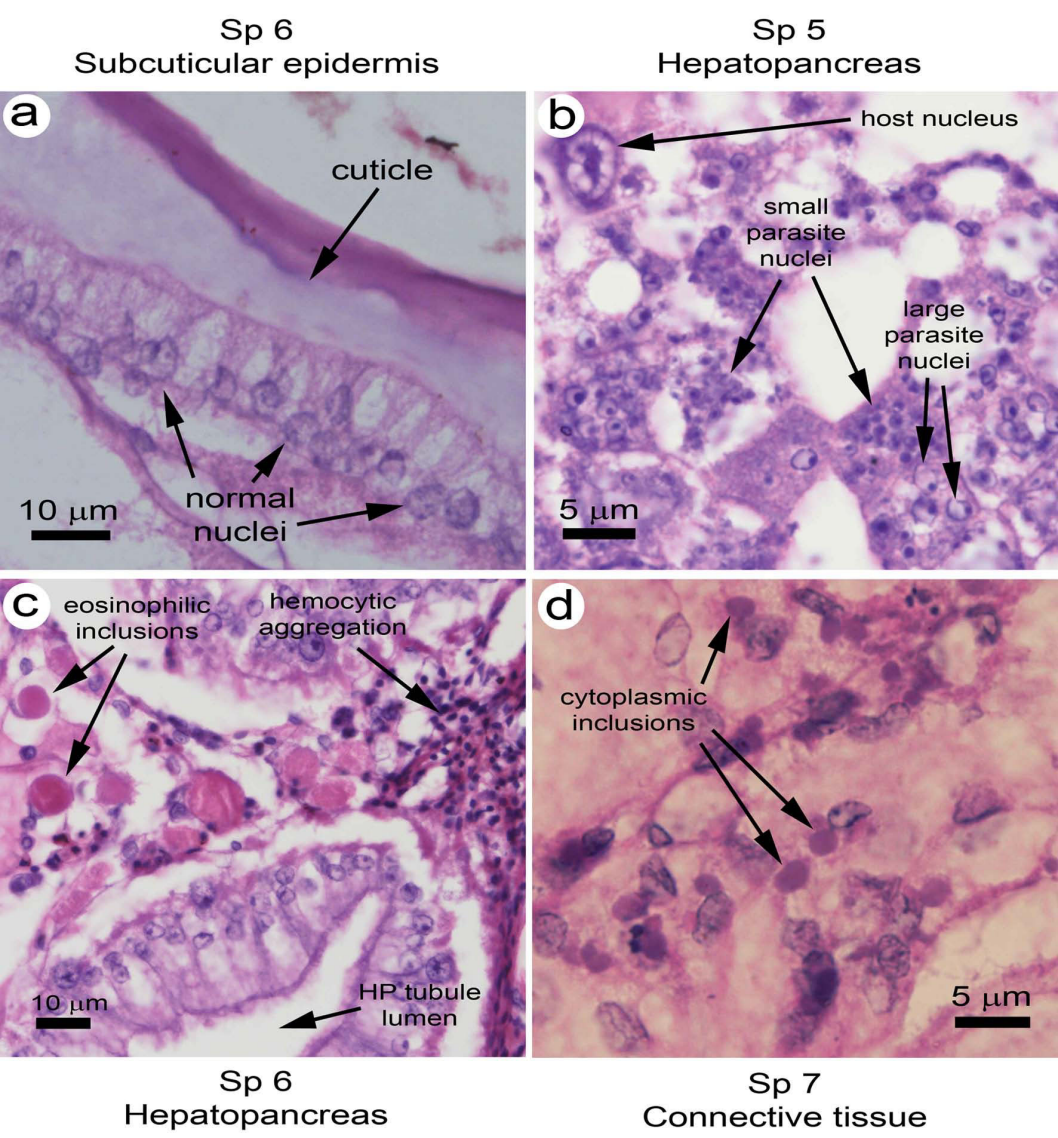
**Histology of *S. paramamosain *challenged with WSSV**. (**a**) Example of normal subcuticular epidermis lacking WSSV lesions and consistent with the negative PCR test kit and real time PCR results. (**b**) Example of a hepatopancreatic tissue lesion resembling those caused by crustacean microsporidians undergoing spore development. (**c**) Example of a hepatopancreatic lesion showing large eosinophilic cytoplasmic inclusions of unknown nature and also hematocytic aggregation typical of severe bacterial infections. (**d**) Example of small cytoplasmic inclusions adjacent to the nuclei of connective tissue cells.

For *S. paramamosain*, the moribund specimens challenged with doses of 5 × 10^5 ^or less gave negative reactions for WSSV infections using the IQ2000 test kit, negative qPCR results (i.e., below the detection limit) and negative histopathology for WSSV lesions (Figure 5a). Of the three specimens that died after the challenge dose of 5 × 10^5 ^(i.e., Sp5, 6, 7) histological examination revealed the presence of severe lesions by other pathogens that may have been the cause of mortality (Figure 5b-d). In Sp5, there were severe lesions in tubule epithelial cells of the hepatopancreas that showed parasite nuclei of large and small sizes, resembling those of a microsporidian undergoing spore development [24] (Figure 5b). In Sp6, the hepatopancreas had severe lesions of a different type, this time in the connective tissue cells showing very large, eosinophilic, viral-like, cytoplasmic inclusions and accompanied by hemocytic aggregation (Figure 5c). In Sp7, the connective tissue of the hepatopancreas, muscle and epidermis showed large numbers of small, magenta, viral-like, cytoplasmic inclusions often adjacent to nuclei (Figure 5d). The severity and number of these lesions were consistent with possible cause of mortality. Hemocytes of these specimens examined by confocal microscopy (as described above) revealed positive immunofluorescence for WSSV in the cytoplasm (Figure 4d).

After the highest challenge dose of 5 × 10^6 ^copies per g, the 2 moribund crab specimens gave IQ2000 test kit results for severe WSSV infections and this corresponded to the qPCR results of 1.2 × 10^9 ^and 8.9 × 10^8 ^WSSV copies per 100 ng total DNA. The 2 remaining crabs that still survived at the end of the experiment (106 days) gave IQ2000 test kit results for a medium infection and a medium-to-severe infection, and the corresponding qPCR results gave 2.9 × 10^3 ^and 5.4 × 10^3 ^WSSV copies per 100 ng total DNA, respectively. None of these high-dose specimens were examined for WSSV histopathology, but the 2 crabs that survived to the end of the experiment (106 days) showed positive immunofluorescence in the cytoplasm of very few hemocytes on day 59 (data not shown) and most of the hemocytes on day 82 (Figure 4d), which was 26 days after the highest dose challenge on day 56. The nuclei of these hemocytes were condensed and often fragmented possibly indicating apoptosis leading to hemocyte depletion as previously reported for shrimp infected with WSSV [25].

Results for *S. paramamosain *are more difficult to interpret than those for *S. olivacea *because all 5 crabs that died at injection doses lower than 1 × 10^6 ^WSSV copies per g gave negative test results for WSSV infection using both the IQ2000 kit and qPCR. If they had died from WSSV, kit reactions for severe WSSV infections and qPCR results indicating high loads of WSSV would have been expected. Histopathological analysis for 3 of the 5 specimens revealed no WSSV lesions but instead severe lesions from at least 3 unknown pathogens that could have been the cause of their death. As with the 2 similar, low-dose challenge *S. olivacea *specimens described above, we assume that these *S. paramamosain *died as a result of some undetermined complication resulting from the WSSV injection, but not because of WSSV alone and not because of the injection process itself, since none of the control crabs died after buffer injections.

Although the *S. paramamosain *specimens did not show typical WSSV lesions with enlarged basophilic nuclei in normal WSSV-target tissues such as the subcuticular epidermis and gills, they did show immunopositive hemocytes, most with condensed or karyorrhectic nuclei indicative of apoptosis as previously reported for WSSV infections in shrimp [25]. In addition, the two specimens (Sp12 and Sp13 representing 22.22% of the crabs) that survived the highest WSSV challenge dose showed WSSV immunopositive hemocytes after challenge on day 56, and the proportion of positive cells was higher on day 87 than on day 59, indicating that viral replication had occurred in the interim. For these specimens, the PCR test kit results were below the severe level and the WSSV loads by qPCR were low. Furthermore, the immunopositive reaction was in the hemocyte cytoplasm only, while WSSV virions are normally assembled in the nucleus. Using these facts to attempt to explain our results, we speculate that hemocytes may be the prime target for WSSV in *S. paramamosain *and that the virus may replicate there, but induce apoptosis before mature virions are assembled. The resulting depletion of hemocytes could impair the host ability to combat pre-existing infections or new infections by other pathogens, and that would explain the variety in the histopathology seen in the moribund *S. paramamosain *we examined. It would also explain why there was no mortality from other infectious agents in the control crabs injected with buffer only. At the same time, it would also suggest that the hemocytes play an important role in the *S. paramamosain *response to WSSV, and that they normally limit or clear WSSV, except when overwhelmed by a sufficient challenge dose. Thus, *S. paramamosain *challenged with WSSV may be a good model organism to study the process of WSSV clearance in crabs.

## Overall comparison

In summary, the results revealed great individual variation in the response of *S. olivacea *and *S. paramamosain *to WSSV. At the same time, both species are susceptible to white spot disease, in contrast with *S. serrata *that has been reported to show no mortality even in the presence of extensive WSSV lesions. In addition, *S. olivacea *was more susceptible to disease than *S. paramamosain*, since its survival probability at 70 days post-infection was 0 while that for *S. paramamosain *was 0.22. Thus, based on previous published work for *S. serrata *and on our results, we propose that susceptibility to white spot disease in the genus *Scylla *is species-dependent but may also be dose-history dependent as seen with *S. olivacea*. In practical terms for shrimp farmers, our results show that *S. olivacea *and *S. paramamosain *may be infected with WSSV for periods of several weeks or more and potentially act as carriers during that interval, but that they might pose less threat than *S. serrata *that is apparently unaffected by WSSV infection. For crab farmers, our results suggest that rearing of *S. serrata *would be a better choice than *S. paramamosain *or *S. olivacea *in terms of avoiding losses from seasonal WSSV outbreaks.

## Competing interests

The authors declare that they have no competing interests.

## Authors' contributions

NS participated in the conception and design of the study, carried out all experimental work, collected and analyzed the data and contributed to writing the manuscript. SM participated in the preliminary *S. olivacea *test. CK participated in the design of the study. KS conceived and participated in the design of the study. TWF did the histopathological analysis of the WSSV-infected crabs and participated in data analysis and manuscript writing. All authors read and approved the final manuscript.
